# Omicron surge impact on acute kidney injury in ICU patients: A study using the ISARIC COVID-19 database

**DOI:** 10.1371/journal.pone.0336843

**Published:** 2025-11-20

**Authors:** Danyang Dai, Pedro Franca Gois, Marina Wainstein, Moji Ghadimi, Nicholas Spyrison, Rolando Claure-Del Granado, Sally Shrapnel, Jason D. Pole, ISARIC Characterization Group

**Affiliations:** 1 Centre for Health Services Research, University of Queensland, Brisbane, Australia; 2 Faculty of Health, Medicine & Behavioural Sciences, University of Queensland, Brisbane, Australia; 3 Nephrology and Transplantation, John Hunter Hospital, New South Wales, Australia; 4 School of Mathematics and Physics, University of Queensland, Brisbane, Australia; 5 Division of Nephrology Hospital Obrero No 2 – Caja Nacional de Salud, Cochabamba, Bolivia; 6 IIBISMED, Universidad Mayor de San Simon, School of Medicine, Cochabamba, Bolivia; 7 ARC Centre of Excellence for Engineered Quantum Systems, School of Mathematics and Physics, University of Queensland, Queensland, Australia; 8 Dalla Lana School of Public Health, The University of Toronto, Toronto, Ontario Canada; 9 International Severe Acute Respiratory and Emerging Infections Consortium (ISARIC), Pandemic Sciences Institute, University of Oxford, Oxford, United Kingdom; Mahidol University, Faculty of Tropical Medicine, THAILAND

## Abstract

**Background:**

Acute Kidney Injury (AKI) is common among COVID-19 patients and is associated with a higher risk of death. Compared to earlier COVID-19 variants, Omicron has reduced mortality. To study the relationship between Omicron and AKI, we conducted the first international study using the global International Severe Acute Respiratory and Emerging Infection Consortium (ISARIC) COVID-19 global dataset.

**Methods:**

This prospective observational study aims to characterise AKI in a cohort of 3,908 COVID-19 patients admitted to the intensive care unit (ICU) across six countries. Clinical characteristics were compared between Omicron and pre-Omicron patients. Multivariable logistic regression was used to analyse the relationship between the Omicron variant and AKI. AKI was defined based on the change in serum creatinine levels, in accordance with the Kidney Disease Improving Global Outcome AKI guidelines.

**Results:**

Patients admitted to an ICU during the Omicron wave were older and had more comorbidities than pre-Omicron patients. The prevalence of AKI was the same between Omicron and previous variants (24.7% vs 22.9%, p-value = 0.321). Controlling for confounders, ICU patients with the Omicron variant were 30%−40% less likely to develop AKI compared to patients with previous variants. The survival curves between AKI patients with Omicron and non-Omicron variants were consistent with the survival analysis.

**Conclusion:**

After adjusting for demographics, comorbidities, laboratory findings, and treatments, patients in ICU during the Omicron wave were less likely to develop AKI compared to previous eras. Nevertheless, the precise influence of the Omicron variant on kidney function remains a subject of ongoing discussion.

## Introduction

SARS-CoV-2 has, thus far, infected more than 769 million people and resulted in the loss of over 6.9 million lives [1]. In November 2021 Omicron (B1.1.529) was identified as a new variant of concern by the World Health Organization (WHO) [2]. Omicron was initially identified in South Africa and showed increased transmissibility [3], but reduced clinical severity and mortality compared to previous variants [3–7]. Acute kidney injury (AKI) has been recognized as one of the most prevalent and critical issues in patients admitted to hospital with COVID-19 [8–11] resulting in extended hospital stays, higher need for intensive care, and in-hospital death [8–11]. The prevalence of AKI among COVID-19 hospitalized patients between January 2020 and August 2020 was estimated to be 19.45% in a systematic review combining 40 studies [12].

Thus far, most of the COVID-19 and AKI studies have been limited to the earlier period of the pandemic, providing limited information about the relationship between Omicron and AKI development [12]. A single-centre study revealed a three-fold higher prevalence of AKI among intensive care unit (ICU) patients infected with the Omicron versus the Delta variant. Another study identified significant higher prevalence of AKI in the 4^th^ wave which corresponds to the Omicron period compared to the 3^rd^ wave [13]. In this study, the 4^th^ wave AKI prevalence was lower compared to the first and second wave which corresponds to the beginning of COVID [13]. As both studies have limited patient cohorts, larger and more diverse population base is needed to further understand this relationship.

To further study the association between the Omicron variant and the prevalence of AKI in the ICU setting, we conducted the first multinational study focusing on AKI among ICU patients using the International Severe Acute Respiratory and Emerging Infections Consortium (ISARIC) COVID-19 database. We examined the prevalence of AKI and the characteristics and outcomes of patients admitted with various COVID-19 viral variants. Based on current literature, we hypothesized that patients infected with the Omicron variant would have a higher prevalence of AKI but a lower prevalence of in-hospital complications and death.

## Methods

### Study design

The findings in this paper utilized the ISARIC COVID-19 global database. This database includes clinically diagnosed or laboratory-confirmed SARS-CoV-2 infection data collected from 76 countries where 1,807 ISARIC partner institutions are located [14]. Since the beginning of the outbreak, the ISARIC-World Health Organisation (WHO) Clinical Characterisation Protocol (CCP) for Severe Emerging Infections has provided a framework for observational and prospective data collection on hospitalised patients using standardized case report forms (CRF) [15]. The protocol, CRF and study information can be found at https://isaric.org/research/covid-19-clinical-research-resources.[8,16,17]. Serum creatinine measurements were collected from all sites without standardizing time intervals, and each site had the freedom to determine the collection frequency [9].

In this prospective observational study, there were no changes made to clinical management practices. The protocol and consent forms can be accessed on the study website (https://isaric.net/ccp/). Given the overwhelming nature of the pandemic, while written consent was obtained in most sites, some sites approved oral consent or waiver of consent [8, 9]. Full details are available on the study website. The ISARIC-WHO CCP obtained approval from the World Health Organization Ethics Review Committee (RPC571 and RPC572, 25 April 2013). Ethical approval was obtained for each participating country and site following local guidance and requirements (Supplementary Statement in S1 File). This study follows the Strengthening the Reporting of Observational Studies in Epidemiology (STROBE) reporting guideline (S1 Table in S1 File).

### Study population

#### Inclusion and exclusion criteria.

We included adult patients (aged 18 + years) from the ISARIC-WHO CCP database who were admitted to Intensive Care Units between June 1, 2021, and October 31, 2022, from countries reporting at least 10% of laboratory values for their patients. Criteria for clinical diagnosis can be found in S2 Table in S1 File. The study excluded non-ICU individuals; those who were identified as being on maintenance kidney replacement therapy (KRT) (dialysis or kidney transplantation); and patients who did not have a specified outcome (death, discharge or transfer). Patients with fewer than two serum creatinine measurements were also excluded from the study given that these were needed to determine AKI. To mitigate biases stemming from limited sample sizes, countries and regions (Brazil, Hong Kong, India, Kuwait, Libya and New Zealand) with fewer than 30 patients for both the pre-Omicron and Omicron periods were excluded.

Patients were categorized into two time periods: “Pre-Omicron” for admissions before December 1, 2021, and “Omicron” for admissions after January 1, 2022. These dates were chosen as they reflect the point at which the Omicron variant frequency reached 90% in the countries included in this study [4]. The exception to this was Pakistan which reported a peak Omicron frequency during mid-January 2022 [4]. December 2021 was considered a period of change in dominance of specific COVID-19 variants, where Omicron was spreading globally and incidence increasing while Delta was waning. As there were no clinical flags in the datasets that identified specific variants, it was not feasible to discriminate patients infected with the Omicron variant from pre-Omicron variants in December 2021 thus these data were excluded.

### AKI

AKI was defined according to the Kidney Disease Improving Global Outcome (KDIGO) guidelines serum creatinine criteria [18]. The KDIGO guidelines require a patient to have an increase in serum creatinine by 26.5 µmol/l within 48 hours or an increase to more than 1.5 times the baseline serum creatinine within 7 days. As most patients only have one reading of serum creatinine within 48 hours, an increase to more than 1.5 times the baseline serum creatinine within 7 days was used to identify AKI. As pre-admission serum creatinine values were unavailable, the first serum creatinine within 48 hours from admission was used as the baseline value [18]. KDIGO urine output criteria were not applied due to the infrequent collection of urine volume information in the CRF. The gradings of AKI were classified according to the KDIGO guidelines. AKI was categorised into community-acquired AKI (CA-AKI) if identified within the first 48 hours of admission and hospital-acquired AKI (HA-AKI) if it developed thereafter [19].

Patients’ details, including comorbidities, medications, signs and symptoms, observations and laboratory test results were collected from the pre-specified CRF. Admission treatments, complications and outcomes were also reported. The outcomes included discharge, transfer to another hospital and in-hospital death. Comprehensive definitions for all recorded variables can be found in S3 Table in S1 File.

### Statistical Analysis

Descriptive statistical analysis was conducted with RStudio 2023.06.1 + 524 [20, 21]. Continuous variables were presented as medians and interquartile ranges (IQR), whereas categorical variables were depicted as counts and percentages. All statistical tests were performed using pairwise independent sample comparisons for the pre-Omicron cohort versus the Omicron cohort. The Mann-Whitney U test was performed for continuous variables and Pearson’s Chi-squared test was used for categorical variables. Standardized mean differences (SMDs) were used to describe the differences between pre-Omicron patients and Omicron patients [22]. The relationship between in-hospital death and AKI is presented using a Kaplan-Meier survival curve and further stratified by viral variant [23]. The follow-up duration was calculated in days, starting from the first day of hospital admission and concluding with the earliest date of discharge, death or transfer to another hospital.

Logistic regression models were estimated to assess the relationship between AKI and the Omicron variant. Variables included in the logistic regression models are presented in Table 2 alongside odds ratios, p-values and confidence intervals for the Omicron variant. Sandwich estimator was adopted to adjust for the standard errors to account for potential correlation in covariates [24]. Variable selection was based on known predictors of AKI from the existing literature [25]. Variables with more than 20% missingness were excluded from the analysis. Missing values for other variables included were imputed using multiple imputations by chained equation [26]. A list of imputed variables can be found in S1 Fig in S1 File. We examined the relationship between AKI and the Omicron variant controlling for demographic variables, comorbidities, laboratory studies and treatments. Age was categorised into 10-year bins starting from 30 years of age, the reference group for the logistic regression is age from 18 to 29. The sex information pertains to the “biological sex assigned or determined at birth”. The models were fitted to data from pre-Omicron and Omicron periods. The pre-Omicron period served as the reference group, allowing for a specific examination of the relationship between the Omicron variant and the risk of AKI.

### Ethics approval and consent to participate

Ethics Committee approval for this work was given by the World Health Organisation Ethics Review Committee (RPC571 and RPC572 on 25 April 2013). Institutional approval was additionally obtained by participating sites including the South Central Oxford C Research Ethics Committee in England (Ref 13/SC/0149) and the Scotland A Research Ethics Committee (Ref 20/SS/0028) for the United Kingdom and the Human Research Ethics Committee (Medical) at the University of the Witwatersrand in South Africa as part of a national surveillance programme (M160667) collectively representing the majority of the data. Other institutional and national approvals are in place as per local requirements.

## Results

**Fig 1:**
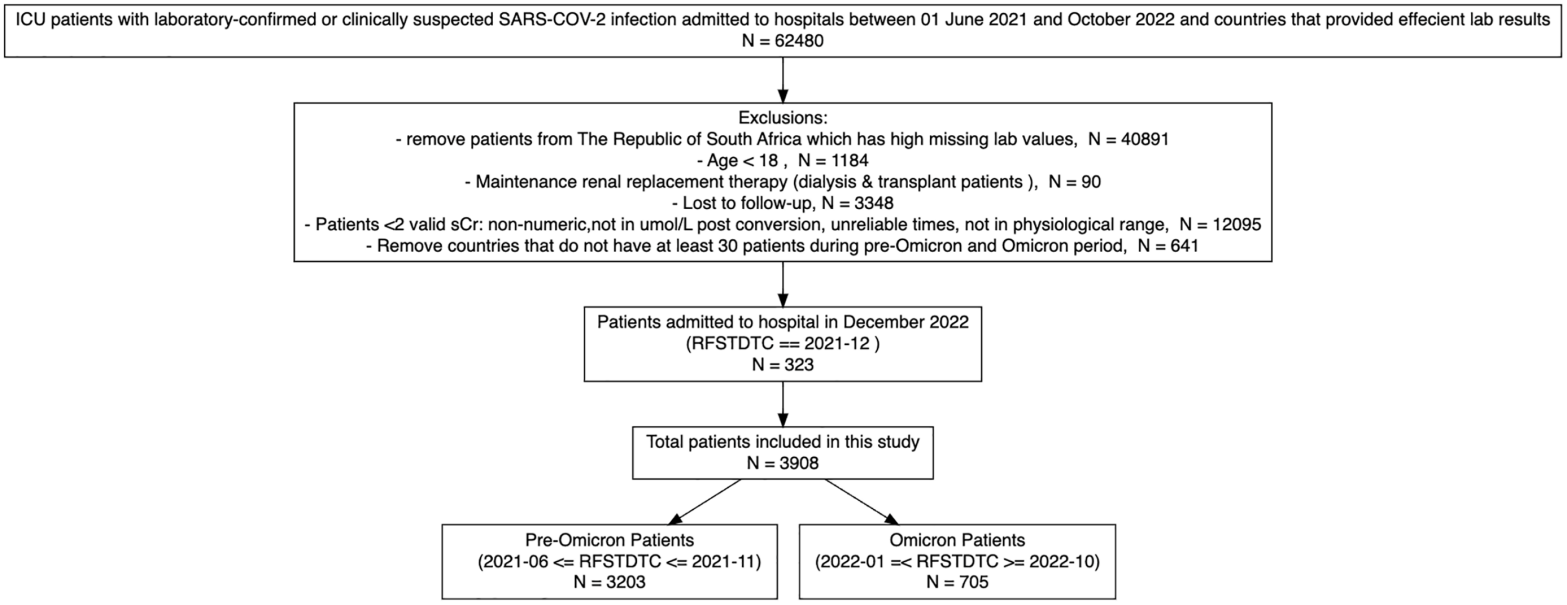
Patients Inclusion and Exclusion Flowchart. ICU: Intensive Care Unit; sCr – serum creatinine; RFSTDTC – Reference Start Date/Time, corresponds with the time and date of the subject’s first study encounter for admission.

**Fig 2:**
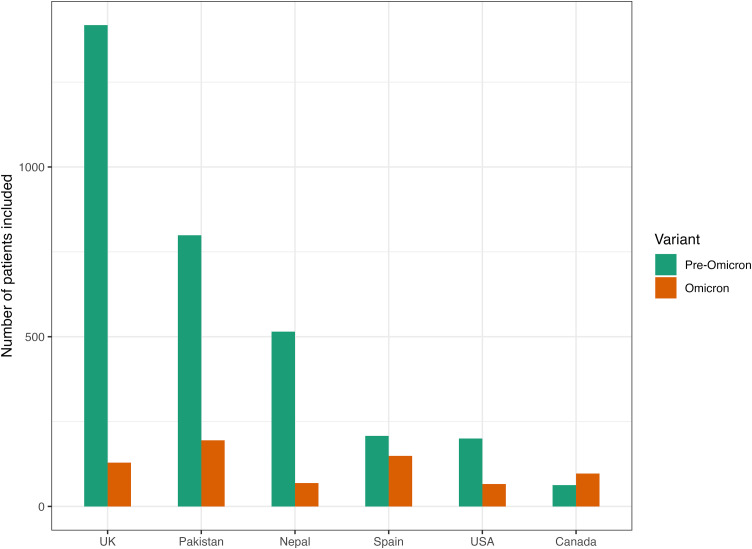
ICU Patients Included in the Study by Countries and Variants. ICU: Intensive Care Unit; USA: United States; UK: United Kingdom.

Data were collected for 62,480 ICU-admitted patients from 47 countries from the 1^st^ of June 2021 to the 31^st^ of October 2022. A breakdown of exclusions can be found in Fig 1. After exclusions 3,908 patients were included in the analysis. Of these, 3,203 were from the pre-Omicron period and 705 were from the Omicron period. The final countries included in this study were Canada, United States (USA), United Kingdom (UK), Spain, Pakistan and Nepal. The distribution of included patients across the six countries is shown in Fig 2. The UK contributed the most patients during the pre-Omicron surge, and Pakistan contributed the highest counts of Omicron cases.

### Demographic and clinical characteristics

Baseline characteristics at hospital admission, acute interventions, complications and outcomes for pre-Omicron and Omicron ICU patients are shown in Table 1. The median age among patients admitted to ICU with COVID-19 pre-Omicron and Omicron variant was 57 (IQR: 45–68) and 63 (IQR: 52–74) years of age respectively. Less than half of the hospitalised patients were female (38% for pre-Omicron and 38% for Omicron). Omicron patients had a higher prevalence of comorbidities compared to pre-Omicron patients. The prevalence of chronic kidney disease was 12% for Omicron patients and 6% for pre-Omicron patients. The median serum creatinine on admission was 76 µmol/L (IQR: 58, 114) for pre-Omicron patients and 87 µmol/L (IQR: 63, 160) for Omicron patients. The median for initial estimated glomerular filtration rate (eGFR) was 88.7 ml/min/1.73m^2^ (IQR: 52.7, 109.1) for pre-Omicron patients and 68.9 ml/min/1.73m^2^ (IQR: 33.3, 97.4) for Omicron patients. Pulmonary symptoms like cough and shortness of breath were significantly less frequent in the Omicron variants than in the pre-Omicron variants (38% vs 64%, p-value < 0.001; 55% vs 75%, p-value < 0.001). Lower usage of invasive mechanical ventilation was observed among Omicron patients compared to pre-Omicron ICU patients (62% vs 72%, p-value < 0.001). Acute dialysis requirement among Omicron and pre-Omicron AKI patients was 45% and 52% respectively (p-value = 0.150).

**Table 1:** Included Patients Characteristics.

	Pre-Omicron				Omicron				Test Statistics between Pre-Omicron and Omicron All Patients	
	All (2 + sCr)	AKI	No AKI	Missing (%)	All (2 + sCr)	AKI	No AKI	Missing (%)	Significant	SMDs
	3203	732	2471		705	174	531			
**Demographics**										
Age, year, median (IQR)	57 (45, 68)	59 (48, 69)	57 (44, 67)	0	63 (52, 74)	62 (50.2, 73)	64 (53, 74)	0	p < 0.001	0.382
Female (%)	1,231 (38)	261 (36)	970 (39)	0.09	268 (38)	57 (33)	211 (40)	0.28	p < 0.001	−0.007
**AKI Grades & RRT, n (%)**										
AKI grade 1	105 (3)	105 (14)	0 (0)	0	29 (4)	29 (17)	0 (0)	0	0.139	0.044
AKI grade 2	89 (3)	89 (12)	0 (0)	0	18 (3)	18 (10)	0 (0)	0	0.704	0.014
AKI grade 3	159 (5)	159 (22)	0 (0)	0	48 (7)	48 (28)	0 (0)	0	0.006	0.078
AKI grade 3 – RRT	379 (12)	379 (52)	0 (0)	0	79 (11)	79 (45)	0 (0)	0	0.565	0.019
Comorbidities**, n (%)										
Chronic Kidney Disease	189 (6)	81 (11)	108 (4)	12.52	83 (12)	29 (17)	54 (10)	3.83	p < 0.001	0.188
Chronic Cardiac Disease	305 (10)	86 (12)	219 (9)	12.43	106 (15)	19 (11)	87 (16)	3.83	p < 0.001	0.141
Chronic Pulmonary Disease	211 (7)	47 (6)	164 (7)	12.21	63 (9)	6 (3)	57 (11)	4.26	0.029	0.066
Hypertension	1,186 (37)	334 (46)	852 (34)	12.11	321 (46)	94 (54)	227 (43)	3.4	p < 0.001	0.101
Diabetes – Type 1&2	452 (14)	84 (11)	368 (15)	53.79	130 (18)	20 (11)	110 (21)	41.56	0.06	0.057
Liver Disease	55 (2)	19 (3)	36 (1)	12.55	44 (6)	7 (4)	37 (7)	3.97	p < 0.001	0.227
Obesity	505 (16)	92 (13)	413 (17)	16.39	94 (13)	19 (11)	75 (14)	8.79	p < 0.001	0.114
**Signs & Symptoms on Admission, n (%)**										
Altered consciousness/confusion	189 (6)	49 (7)	140 (6)	13.49	67 (10)	11 (6)	56 (11)	27.66	p < 0.001	0.212
Diarrhoea	448 (14)	83 (11)	365 (15)	12.33	46 (7)	16 (9)	30 (6)	27.94	p < 0.001	0.210
Fever	2,074 (65)	477 (65)	1,597 (65)	10.52	269 (38)	78 (45)	191 (36)	26.95	p < 0.001	0.425
Vomiting/nausea	418 (13)	68 (9)	350 (14)	12.46	52 (7)	12 (7)	40 (8)	27.66	p < 0.001	0.143
Muscle aches/joint pain	480 (15)	87 (12)	393 (16)	15.99	60 (9)	11 (6)	49 (9)	29.5	p < 0.001	0.162
Headache	266 (8)	33 (5)	233 (9)	15.8	37 (5)	4 (2)	33 (6)	29.5	0.020	0.086
Cough	2,045 (64)	427 (58)	1,618 (65)	9.71	267 (38)	65 (37)	202 (38)	27.23	p < 0.001	0.391
Shortness of breath	2,388 (75)	540 (74)	1,848 (75)	8.71	391 (55)	108 (62)	283 (53)	25.53	p < 0.001	0.175
**Observations on Admission, median (IQR)**										
Temperature, C	37.2 (36.6, 38)	36.9 (36.4, 37.6)	37.3 (36.7, 38.1)	32.63	36.7 (36.3, 37.2)	36.6 (36.2, 37.2)	36.8 (36.3, 37.2)	39.15	p < 0.001	−0.526
Systolic BP, mmHg	127 (113, 140)	129 (114, 141)	126 (113, 140)	3.4	128 (110, 144)	130 (110, 145)	127 (110, 144)	8.51	p < 0.001	0.041
Diastolic BP, mmHg	75 (66, 83)	75 (65, 86)	75 (67, 82)	3.9	72 (60, 83)	70 (60, 84)	73 (61, 82.5)	9.36	p < 0.001	−0.158
Heart rate, BPM	93 (82, 109)	96 (82.5, 110)	93 (82, 108)	3.18	94 (79, 109)	95 (85, 109.2)	92 (78, 109)	6.81	p < 0.001	−0.047
Respiratory rate, per min	24 (20, 30)	26 (21, 31)	24 (20, 30)	6.84	22 (20, 28)	24 (20, 29)	22 (20, 28)	20.28	p < 0.001	−0.267
Oxygen saturation, %	93 (89, 96)	92 (88, 96)	93 (90, 96)	7.68	95 (91, 97)	95 (91, 96)	95 (91, 97)	11.21	p < 0.001	0.268
**Laboratory Results on Admission, median (IQR)**										
Potassium (mmol/L)	4.1 (3.7, 4.5)	4.2 (3.8, 4.7)	4.1 (3.7, 4.4)	2.31	4.2 (3.8, 4.6)	4.2 (3.8, 5)	4.2 (3.8, 4.6)	0.71	p < 0.001	0.197
Serum Creatinine (umol/L)	76 (58, 114)	196.5 (99, 367.2)	69 (54.8, 89)	0	87 (63, 160)	267 (133.1, 362.4)	76 (60, 110.5)	0	p < 0.001	0.129
Serum Creatinine (umol/L) – CKD	151 (102, 317)	363 (181.2, 548)	111.7 (88, 151)	0	163 (108.9, 269.6)	318.2 (194.5, 443)	134.8 (97.5, 173.5)	0	0.931	−0.145
eGFR (ml/min/1.73m2)	88.7 (52.7, 109.1)	27 (13.1, 63.7)	95.8 (72.7, 112.3)	0	68.9 (33.3, 97.4)	21.2 (13.6, 47.5)	82 (52, 102.8)	0	p < 0.001	−0.328
Hemoglobin (g/L)	133 (118, 146)	124 (106, 140)	135 (121, 148)	5.15	123 (105, 139)	113 (96, 132)	127 (109.5, 143)	20	p < 0.001	−0.389
Sodium (mmol/L)	136 (133, 140)	137 (134, 141)	136 (133, 139)	2.78	137 (134, 140)	138 (135, 142)	137 (134, 140)	1.28	p < 0.001	0.107
Platelets (10^9/L)	200 (153, 261.8)	199 (149, 269.5)	200 (154, 259)	12.89	220 (168, 291)	205 (164.5, 279.5)	227.5 (168, 292.8)	15.32	p < 0.001	0.188
WBC (10^9/L)	8.1 (5.6, 11.9)	10.9 (6.8, 15.6)	7.7 (5.5, 11)	18.51	9.6 (6.5, 14.2)	10.2 (7, 15.1)	9.4 (6.3, 13.6)	10.64	p < 0.001	0.251
**In hospital Treatment, n (%)**										
Antiviral and COVID-19 targeting agents	1,568 (49)	357 (49)	1,211 (49)	0.78	288 (41)	85 (49)	203 (38)	1.13	p < 0.001	0.162
Antibiotic agents	2,865 (89)	707 (97)	2,158 (87)	0.56	606 (86)	164 (94)	442 (83)	0.99	p < 0.001	0.098
Antifungal agents	332 (10)	79 (11)	253 (10)	3.28	77 (11)	18 (10)	59 (11)	1.13	0.755	0.011
Corticosteroids	2,836 (89)	620 (85)	2,216 (90)	0.28	517 (73)	146 (84)	371 (70)	0.85	p < 0.001	0.388
Invasive mechanical ventilation	2,311 (72)	668 (91)	1,643 (66)	0.31	434 (62)	138 (79)	296 (56)	0.14	p < 0.001	0.230
vasopressors	1,162 (36)	504 (69)	658 (27)	2.59	252 (36)	93 (53)	159 (30)	1.56	0.535	0.019
Complications**, n (%)										
Bacterial pneumonia	413 (13)	88 (12)	325 (13)	12.39	80 (11)	13 (7)	67 (13)	7.8	0.028	0.071
Cardiac arrest	209 (7)	91 (12)	118 (5)	8.68	66 (9)	25 (14)	41 (8)	4.11	0.001	0.094
Coagulation disorder	123 (4)	26 (4)	97 (4)	11.36	21 (3)	6 (3)	15 (3)	7.8	0.085	0.058
Rhabdomyolysis	10 (0)	6 (1)	4 (0)	11.49	2 (0)	0 (0)	2 (0)	8.51	1.000	0.007
**Outcomes, n (%)**										
Transferred	216 (7)	30 (4)	186 (8)	0	51 (7)	12 (7)	39 (7)	0	0.547	0.019
Discharged	1,501 (47)	117 (16)	1,384 (56)	0	410 (58)	55 (32)	355 (67)	0	p < 0.001	0.228
Death	1,486 (46)	585 (80)	901 (36)	0	244 (35)	107 (61)	137 (26)	0	p < 0.001	0.242
Length of Stay (median, IQR)	11 (6, 18)	9 (5, 17)	11 (7, 19)	0.91	10 (5, 19)	7 (4, 13.2)	11 (5, 21)	0.71	p < 0.001	0.017

eGFR: estimated glomerular filtration rate,sCr: serum creatinine, AKI: Acute Kidney Injury, SMD: standardized mean difference, WBC: white blood count

**: Comprehensive definitions for comorbidities, complication and outcomes can be found in S3 Table in S1 File

### Outcomes

**Fig 3:**
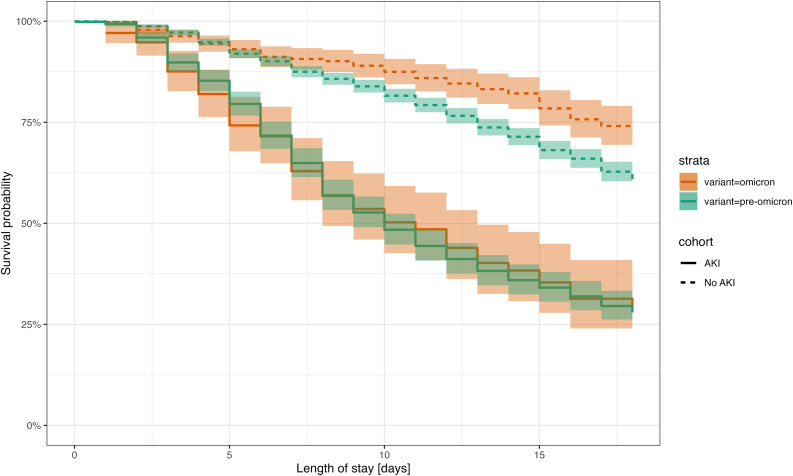
Kaplan–Meier Survival plot stratified across acute kidney injury and variant. Confidence bars are used to illustrate a 95% confidence interval. AKI, acute kidney injury.

In-hospital mortality was 46% for pre-Omicron ICU patients and 35% for Omicron ICU patients. The median time for ICU hospital stay was 11 (IQR: 6–18) days for pre-Omicron patients and 10 (IQR: 5–19) days for Omicron patients. The Kaplan-Meier curve depicted in Fig 3 indicated that AKI patients exhibit lower survival rates in comparison to non-AKI patients (log-rank test: p-value < 0.001). Among AKI patients, there was no difference between Omicron patients and pre-Omicron patients (Fig 3). The log-rank tests indicated no statistical difference between Omicron and pre-Omicron AKI patients for survival curves (log-rank test: p-value = 0.200). For non-AKI patients, the Kaplan-Meier curve suggests that Omicron patients have a higher survival rate compared to pre-Omicron patients (log-rank test: p-value < 0.001).

### AKI and logistic regression

**Fig 4:**
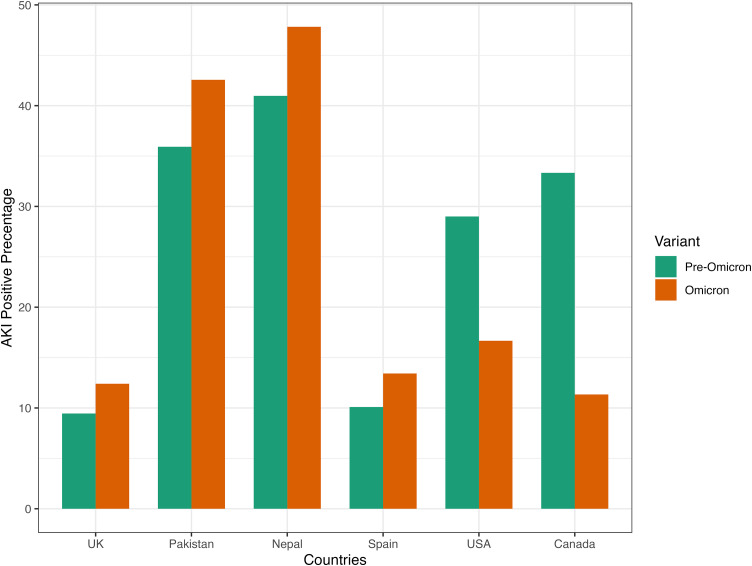
AKI Positive Percentage by Countries and Variants. AKI: acute kidney injury; USA: United States; UK: United Kingdom.

**Fig 5:**
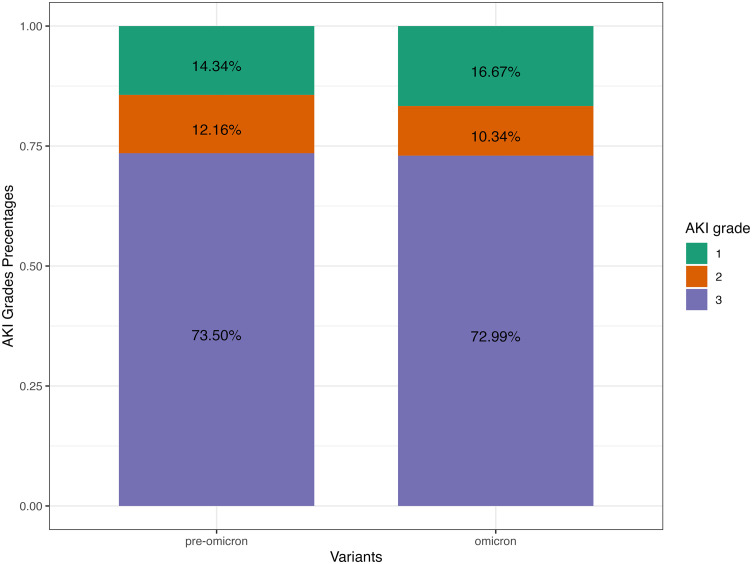
AKI Grades by variants, AKI are graded according to KDIGO guideline. AKI: acute kidney injury; KDIGO: Kidney Disease Improving Global Outcome.

**Fig 6:**
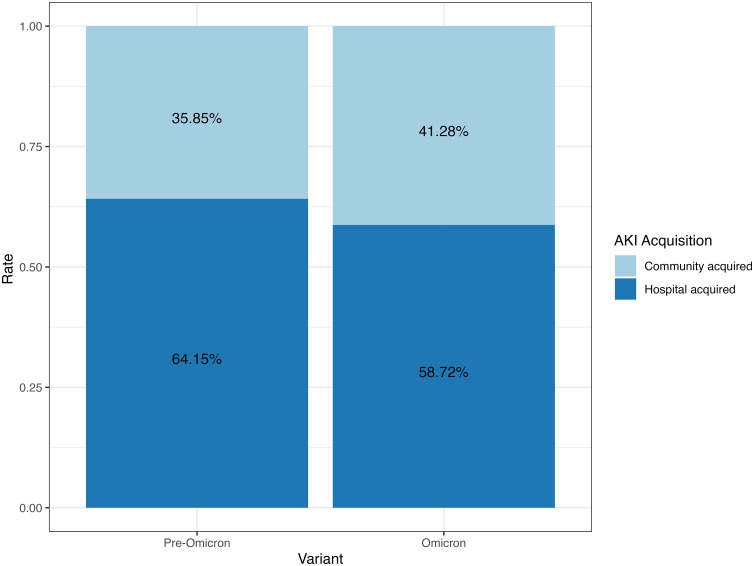
Community-acquired versus hospital-acquired acute kidney injury based on 48 hour cut-off from admission by variants. AKI, acute kidney injury.

For pre-Omicron periods, 732 (22.85%) patients were observed to have developed AKI. During the Omicron surge, 174 (24.68%) out of 705 Omicron patients were observed to have developed AKI. There was no statistical difference in the prevalence of AKI between pre-Omicron and Omicron ICU patients (p-value = 0.321). There were no statistical differences for stage 1 and stage 2 AKI between pre-Omicron and Omicron ICU patients (p-value = 0.139 and p-value = 0.704, respectively). There was a higher prevalence of stage 3 AKI patients observed in Omicron surge compared to pre-Omicron (p-value = 0.006). Fig 4 shows the AKI positive percentage breakdown in different countries. The lower-income countries (Pakistan and Nepal) had consistently higher AKI prevalence for pre-Omicron and Omicron periods compared to other countries (Fig 4). The USA and Canada had a lower AKI prevalence during the Omicron period (Fig 4). Most ICU patients with AKI were classified as AKI grade 3, accounting for 73.5% during the pre-Omicron period and 73.0% during the Omicron period (Fig 5). There was a consistent distribution of AKI grades among ICU patients between the pre-Omicron and Omicron periods (Fig 5). The chi-squared test between AKI grades and variant has shown no difference between the AKI grades between the pre-Omicron variant and Omicron variant (p-value = 0.635). Additionally, more than half of AKI patients experienced hospital-acquired AKI (HA-AKI) regardless of the viral variant, as shown in Fig 6. Table 2 presents the findings from multivariable logistic models addressing the association between Omicron and AKI. Multiple models were fit to include different control variables with AKI positive as the outcome in each model. Using the imputed data, Model 1 and Model 2 suggest that controlling for demographics variables and initial serum creatinine, Omicron patients admitted to ICU were more than 30% less likely to develop AKI. While after additionally controlling for comorbidities, lab results and treatments, there was no significant effect on whether the ICU patients had the Omicron or pre-Omicron variant on AKI.

**Table 2:** Multivariable Logistic Regression Outputs: Association between Omicron and AKI.

Logistic Regression	Data 1 Imputed Data	Model 1			Model 2			Model 3		
	*Predictors*	*Odds Ratios*	*CI*	*p*	*Odds Ratios*	*CI*	*p*	*Odds Ratios*	*CI*	*p*
	(Intercept)	0.03	0.03–0.04	**<0.001**	0.04	0.02–0.07	**<0.001**	0	0.00–0.00	**<0.001**
	Omicron	0.66	0.50–0.87	**0.004**	0.69	0.50–0.94	**0.019**	0.78	0.56–1.09	0.142
	Serum Creatinine	1.02	1.02–1.02	**<0.001**	1.02	1.02–1.02	**<0.001**	1.02	1.02–1.02	**<0.001**
Demographics	Age (30–39)				0.62	0.38–1.00	0.051	0.66	0.39–1.09	0.104
	Age (40–49)				0.85	0.55–1.32	0.466	0.89	0.56–1.41	0.623
	Age (50–59)				0.77	0.50–1.17	0.219	0.74	0.47–1.17	0.195
	Age (60–69)				0.59	0.39–0.90	**0.013**	0.60	0.38–0.95	**0.028**
	Age (70–79)				0.33	0.21–0.52	**<0.001**	0.39	0.24–0.64	**<0.001**
	Age (80–89)				0.42	0.24–0.71	**0.001**	0.59	0.33–1.06	0.079
	Age (>90)				0.08	0.02–0.37	**0.001**	0.08	0.01–0.45	**0.004**
	Spain				1.04	0.54–2.01	0.903	2.33	1.13–4.81	**0.022**
	The UK				0.46	0.25–0.85	**0.013**	1.05	0.55–1.98	0.892
	Nepal				2.89	1.57–5.31	**0.001**	3.71	1.90–7.22	**<0.001**
	Pakistan				2.03	1.13–3.65	**0.017**	3.73	1.95–7.14	**<0.001**
	The US				1.31	0.68–2.53	0.414	1.57	0.80–3.09	0.190
	Female				1.25	1.01–1.55	**0.038**	1.26	0.99–1.59	0.055
Comorbidities**	Liver Disease							1.06	0.56–2.02	0.857
	Hypertension							0.84	0.66–1.06	0.144
	Chronic Pulmonary Disease							1.31	0.85–2.02	0.226
	Chronic Kidney Disease							1.25	0.79–1.99	0.338
	Chronic Cardiac Disease							0.92	0.63–1.35	0.666
	Diabetes							0.99	0.69–1.42	0.969
Lab Value	Hemoglobin							1.00	0.99–1.00	0.443
	Potassium							1.1	0.93–1.30	0.274
	Platelets							1.00	1.00–1.00	0.443
	WBC							1.03	1.01–1.05	**0.006**
	Sodium							1.02	1.00–1.04	0.053
Treatment	Mechanical Ventilation							1.98	1.39–2.83	**<0.001**
	Antibiotic							2.33	1.42–3.81	**0.001**
	Vasopressors							3.30	2.60–4.20	**<0.001**
Observations		3903			3903			3903		
R2 Tjur		0.420			0.464			0.506		
Logistic Regression	Data 2 Listwise deletion	Model 1			Model 2			Model 3		
	*Predictors*	*Odds Ratios*	*CI*	*p*	*Odds Ratios*	*CI*	*p*	*Odds Ratios*	*CI*	*p*
	(Intercept)	0.03	0.03–0.04	**<0.001**	0.04	0.02–0.07	**<0.001**	0.00	0.00–0.00	**<0.001**
	Omicron	0.66	0.50–0.87	**0.004**	0.69	0.50–0.94	**0.019**	0.35	0.20–0.62	**<0.001**
	Serum Creatinine	1.02	1.02–1.02	**<0.001**	1.02	1.02–1.02	**<0.001**	1.02	1.02–1.02	**<0.001**
Demographics	Age (30–39)				0.62	0.38–1.00	0.051	0.90	0.38–2.13	0.818
	Age (40–49)				0.85	0.55–1.32	0.466	1.01	0.46–2.19	0.983
	Age (50–59)				0.77	0.50–1.17	0.219	0.77	0.34–1.75	0.53
	Age (60–69)				0.59	0.39–0.90	**0.013**	0.62	0.28–1.34	0.221
	Age (70–79)				0.33	0.21–0.52	**<0.001**	0.44	0.19–1.02	0.054
	Age (80–89)				0.42	0.24–0.71	**0.001**	0.73	0.28–1.95	0.534
	Age (>90)				0.08	0.02–0.37	**0.001**	0.15	0.01–1.57	0.113
	Spain				1.04	0.54–2.01	0.903	0.88	0.27–2.84	0.826
	The UK				0.46	0.25–0.85	**0.013**	0.78	0.36–1.69	0.531
	Nepal				2.89	1.57–5.31	**0.001**	3.31	0.79–13.74	0.100
	Pakistan				2.03	1.13–3.65	**0.017**	2.27	1.02–5.05	**0.044**
	The US				1.31	0.68–2.53	0.414	1.26	0.59–2.70	0.555
	Female				1.25	1.01–1.55	**0.038**	1.17	0.81–1.68	0.406
Comorbidities**	Liver Disease							1.72	0.74–3.98	0.207
	Hypertension							0.82	0.56–1.19	0.297
	Chronic Pulmonary Disease							1.20	0.60–2.40	0.608
	Chronic Kidney Disease							1.18	0.62–2.23	0.618
	Chronic Cardiac Disease							1.01	0.62–1.66	0.957
	Diabetes							0.99	0.61–1.61	0.966
Lab Value	Hemoglobin							1.00	0.99–1.01	0.835
	Potassium							0.95	0.70–1.28	0.723
	Platelets							1.00	1.00–1.00	0.635
	WBC							1.01	0.98–1.04	0.504
	Sodium							1.04	1.01–1.07	**0.017**
Treatment	Mechanical Ventilation							2.24	1.23–4.09	**0.008**
	Antibiotic							4.28	1.77–10.37	**0.001**
	Vasopressors							2.86	1.92–4.28	**<0.001**
Observations		3903			3903			1863		
R2 Tjur		0.420			0.464			0.522		
Logistic Regression	Data 3 Imputed Data without Spain and Nepal	Model 1			Model 2			Model 3		
	*Predictors*	*Odds Ratios*	*CI*	*p*	*Odds Ratios*	*CI*	*p*	*Odds Ratios*	*CI*	*p*
	(Intercept)	0.02	0.02–0.03	**<0.001**	0.04	0.02–0.09	**<0.001**	0.00	0.00–0.00	**<0.001**
	Omicron	0.58	0.41–0.83	**0.003**	0.51	0.35–0.76	**0.001**	0.56	0.37–0.83	**0.005**
	Serum Creatinine	1.02	1.02–1.02	**<0.001**	1.02	1.02–1.02	**<0.001**	1.02	1.02–1.02	**<0.001**
Demographics	Age (303132333435363738–39)				0.64	0.36–1.11	0.113	0.67	0.36–1.23	0.197
	Age (40–49)				0.83	0.50–1.39	0.485	0.93	0.53–1.63	0.807
	Age (50–59)				0.75	0.46–1.25	0.271	0.81	0.46–1.42	0.454
	Age (60–69)				0.57	0.35–0.94	**0.027**	0.62	0.35–1.09	0.098
	Age (70–79)				0.25	0.14–0.44	**<0.001**	0.32	0.17–0.60	**<0.001**
	Age (80–89)				0.54	0.28–1.02	0.056	0.88	0.43–1.82	0.730
	Age (>90)				0.12	0.02–0.76	**0.025**	0.09	0.01–1.06	0.056
	Spain									
	The UK				0.41	0.22–0.76	**0.005**	1.01	0.52–1.97	0.983
	Nepal									
	Pakistan				1.83	1.01–3.33	**0.047**	3.54	1.79–7.01	**<0.001**
	The US				1.19	0.61–2.32	0.6	1.47	0.73–2.97	0.277
	Female				1.19	0.92–1.54	0.176	1.25	0.94–1.67	0.123
Comorbidities**	Liver Disease							1.24	0.57–2.69	0.581
	Hypertension							0.73	0.54–0.97	**0.031**
	Chronic Pulmonary Disease							1.14	0.64–2.03	0.650
	Chronic Kidney Disease							1.26	0.74–2.15	0.393
	Chronic Cardiac Disease							1.00	0.66–1.53	0.998
	Diabetes							1.03	0.70–1.52	0.873
Lab Value	Hemoglobin							1.00	0.99–1.01	0.978
	Potassium							1.06	0.85–1.31	0.608
	Platelets							1.00	1.00–1.00	0.462
	WBC							1.02	1.00–1.05	0.053
	Sodium							1.05	1.02–1.07	**<0.001**
Treatment	Mechanical Ventilation							2.53	1.58–4.06	**<0.001**
	Antibiotic							3.24	1.79–5.83	**<0.001**
	Vasopressors							3.53	2.62–4.76	**<0.001**
Observations		2962			2962			2962		
R2 Tjur		0.434			0.475			0.526		

WBC: white blood count

Baseline definition: Age (18–29), country – Canada

**: Comprehensive definitions for comorbidities, complication and outcomes can be found in S3 Table in S1 File

Sensitivity analysis was carried out to examine the regressions. Using the listwise deletion method [27], model 1 and model 2 indicate consistent estimation compared to imputed values. Model 3, however, suggests a 60% decrease in the likelihood of developing AKI for the Omicron variant compared to the pre-Omicron variant. Looking into the imputed values which include lab values, all were within the correct range. When examining the occurrences of the missing values, Nepal had more than 99% missing in white blood cell count (WBC) and Spain had 78% missing values in haemoglobin (HGB) (S-Table 2). To adjust for the noise introduced by imputing large amounts of WBC and HGB values for Nepal and Spain, models were fit using the imputed data excluding Nepal and Spain. Using imputed data without Nepal and Spain, model 3 suggests that controlling for initial serum creatinine, demographics, comorbidities, lab results and treatments, patients admitted to the critical care unit with Omicron variant were more than 40% less likely to develop AKI compared to pre-Omicron variant.

## Discussion

This study is the first global investigation of the relationship between the Omicron variant and the characteristics and outcomes of hospitalized individuals with AKI and COVID-19 in the ICU setting. Since most COVID-19 AKI studies had focused on the onset of the COVID-19 pandemic, the prevalence of complications, such as AKI, associated with the Omicron variant has not been extensively studied. Our study encompassed a significantly diverse ICU population utilizing the ISARIC global COVID-19 dataset, with a total of 3,908 observations across six countries, allowing us to analyse the relationship between Omicron and AKI from a multinational standpoint. This multicentre global study has an overall similar prevalence of AKI among patients between the Omicron variant and Pre-Omicron variant. We observed a lower mortality, a lower prevalence of pulmonary embolism and a higher number of comorbidities among the Omicron variant in relation to previous variants for both AKI and no AKI patients. The logistic regression for AKI using the listwise deletion method and imputed data results in different odds ratio for Omicron and pre-Omicron variants. However, all models suggest a decrease in the likelihood of developing AKI for Omicron patients when controlling for initial serum creatinine, demographics, comorbidities, lab results and treatments.

Omicron infection has been established as having milder clinical characteristics and rapid transmission rates [4, 28]. Omicron variants have also been shown to have a lower risk of hospitalisation and reduced severity [29, 30]. Moreover, there has been a reduction in ICU admission during the Omicron wave compared to previous variants [30, 31]. The current understanding of AKI prevalence in Omicron patients is based on findings from two single-centre studies conducted in Italy and China. The Italian study reported 38.24% of ICU patients with Omicron developed AKI [32]. The Chinese study reported one AKI patient for Omicron hospitalised patients and no AKI patient was recorded for Delta patients [33]. Within our own cohort of ICU patients, we observed that the prevalence of AKI among those with Omicron was 24.68%. Single-centre studies can be restricted in their conclusion due to limited observation and unique regions. Some studies have reported that Omicron hospitalised patients were younger compared to previous variants [29, 33, 34], while others found patients were older [4, 32]. This is consistent with our study where Omicron patients were older. While many studies have established that Omicron is a milder version of COVID-19, the data were often limited to assessing the development of AKI in the context of emerging variants. There have been many studies have explored the risk factors associated with AKI development and COVID-19. One study in the USA has previously developed and validated a model to predict AKI in COVID-19 patients admitted to hospitals during the Delta variant period. Their recent study evaluated the same predictive model for HC-AKI using Omicron variant patients’ data [35]. This study finds that the predictive model controlling for demographics, comorbidities, lab values and routine care can accurately predict the development of HC-AKI across several dominant variants strains [35]. This indicates that the etiology and risk factors for AKI have not substantially changed across these variants for hospital-acquired AKI patients [35]. Based on these findings, our study effectively examines the relationship between the Omicron variant and AKI patients. After controlling for confounders including demographics, comorbidities, lab results and treatments, there was a lower likelihood of AKI among Omicron patients compared to pre-Omicron patients holding other variables constant.

We acknowledge there are a few limitations to this study. First, the ISARIC data collection relied on the collection of information during an evolving pandemic. Consequently, some health information was not recorded due to the limited ability and capacity of healthcare workers and institutions to gather data. The ISARIC dataset includes standard clinical information for patients who were admitted with suspected or confirmed COVID-19, it is important to note that the reasons for their hospitalization have not been consistently recorded [36, 37]. Since the symptoms were moderate for the Omicron variant, some hospitalized patients might have been admitted with non-COVID conditions but subsequently tested positive for COVID-19. Therefore, we were unable to determine whether factors other than COVID-19 may have played a more significant role in the development of AKI. With the limited capacity of data collection during the pandemic, there was no genetic sequencing available to identify specific COVID-19 variant for each patient. Thus, we relied on the admission date where Omicron variant frequency reached 90% in the countries we included [4]. While the 90% threshold provides a strong indication, it does not eliminate the residual risk of including patients infected with variants other than Omicron. Given the ISARIC data was de-identified and no patient-level linkage across institutions, hospital transfers could not be tracked. Second, other confounders may not have been captured in this analysis including vaccination status. According to the COVID-19 vaccinations dashboard, both the UK and Spain, the two dominant countries in this analysis by patient volume, had the highest vaccine doses administered during the Omicron wave [38]. The impact of COVID-19 vaccination in preventing COVID-associated AKI is not yet clearly understood. Further study is needed to account for the vaccination status when investigating the relationship between COVID-19 variants and AKI. Third, as pre-admission creatinine levels were not available, the baseline value chosen for AKI diagnosis using the KDIGO standard was the patients’ first serum creatinine within 48 hours of admission. This approach may result in an underestimation of AKI prevalence as some patients could have already experienced kidney dysfunction before hospitalisation [39]. As precise time of AKI was not recorded, we could not reliably ascertain time-to-event information for all patients. Given this analysis required multiple serum creatinine values to assess AKI, and many subjects that were missing multiple values of serum creatinine, we examined in a multivariable model the relationship between baseline characteristics comparing subjects included and those excluded due to missing multiple serum creatinine. Among baseline characteristics, there were no differences of note apart from subjects (1) from the omicron period and (2) discharged from hospital which decreased the likelihood of inclusion (see S4 Table in S1 File). We estimate these differences would have little potential to bias the current analysis given these relationships were expected.

This study includes six countries that satisfied the selection criteria to provide a statistically reasonable sample size from pre-Omicron and Omicron variants and controls for socioeconomic factors. This implies that generalisability needs to be done with caution and might be an issue when interpreting the results.

To our understanding, this study represents the initial attempt to thoroughly investigate the relationship between the Omicron variant and the consequences of individuals experiencing AKI and COVID-19 in the ICU setting. To the best of our knowledge, our research population constitutes the most extensive multinational group of COVID-19 ICU patients. Omicron patients admitted to ICU were older and had more comorbidities compared to pre-Omicron patients. Mortality was lower among Omicron ICU patients compared with pre-Omicron ICU patients. While the prevalence of AKI was higher in Omicron patients, when controlling for initial serum creatinine, demographics, comorbidities, lab results and treatments, the logistics regression suggests that patients admitted to the hospital with the Omicron variant had a lower risk of developing AKI than the pre-Omicron variant. As the sensitivity analysis between imputed data and listwise deletion showed different odds ratio for Omicron variant, the exact impact of Omicron on kidney function is not certain. The distribution of AKI grades was similar between pre-Omicron and Omicron surge. The relatively unnoticed occurrence of AKI in the Omicron variant brings forth adverse consequences, emphasizing the critical necessity of vigilant monitoring and prompt interventions. With the latest discovery of Omicron EG.5 [40], the challenge of understanding COVID-19 and AKI continues. Further studies are needed to better comprehend the viruses that have changed our lives in many ways and prepare us for future variants and waves of COVID-19.

## Supporting information

S1 Data Omicron impact on AKI Collaborators Affiliations. (XLSX)

S1 File Supplementary file for tables and figures. (DOCX)

## Abbreviation:

AKI: Acute Kidney Injury
CA-AKI: Community-acquired AKI
CCP: Clinical Characterisation Protocol
CRF: Case Report Forms
eGFR: Estimated glomerular filtration rate
HA-AKI: Hospital-acquired AKI
ICU: Intensive Care Unit
IQR: Interquartile ranges
KRT: Kidney Replacement Therapy
SARS-Cov-2: Severe acute respiratory syndrome coronavirus 2
SMDs: Standardized mean differences
STROBE: Strengthening the Reporting of Observational Studies in Epidemiology
WHO: World Health Organization.
