# Correction: Omicron surge impact on acute kidney injury in ICU patients: A study using the ISARIC COVID-19 database

**DOI:** 10.1371/journal.pone.0340016

**Published:** 2025-12-31

**Authors:** Danyang Dai, Pedro Franca Gois, Marina Wainstein, Moji Ghadimi, Nicholas Spyrison, Rolando Claure-Del Granado, Sally Shrapnel, Jason D. Pole, ISARIC Characterization Group

The sixth author’s initials appear incorrectly in the citation. The correct citation is: Dai D, Gois PF, Wainstein M, Ghadimi M, Spyrison N, Claure-Del Granado R, et al. (2025) Omicron surge impact on acute kidney injury in ICU patients: A study using the ISARIC COVID-19 database. PLoS One 20(11): e0336843. https://doi.org/10.1371/journal.pone.0336843

The ORCID iD is missing for the sixth author. Please see the author’s ORCID iD here:

- Author Rolando Claure-Del Granado’s ORCID iD is: 0000-0002-8163-3779 (https://orcid.org/0000-0002-8163-3779).
