# Supplementary material for: Omicron surge impact on acute kidney injury in ICU patients: A study using the ISARIC COVID-19 database
Source: PLoS One. 2025 Nov 20;20(11):e0336843. doi: 10.1371/journal.pone.0336843 (PMC12633887; doi:10.1371/journal.pone.0336843)
Supplement: S1 File — (DOCX) [file pone.0336843.s002.docx]

Supplementary Statement

Statement S1. Study ethics approval

Ethics Committee approval for this work was given by the World Health Organisation Ethics Review Committee (RPC571 and RPC572 on 25 April 2013). Institutional approval was additionally obtained by participating sites including the South Central Oxford C Research Ethics Committee in England (Ref 13/SC/0149) and the Scotland A Research Ethics Committee (Ref 20/SS/0028) for the United Kingdom and the Human Research Ethics Committee (Medical) at the University of the Witwatersrand in South Africa as part of a national surveillance programme (M160667) collectively representing the majority of the data. Other institutional and national approvals are in place as per local requirements.

Supplementary Figure 1


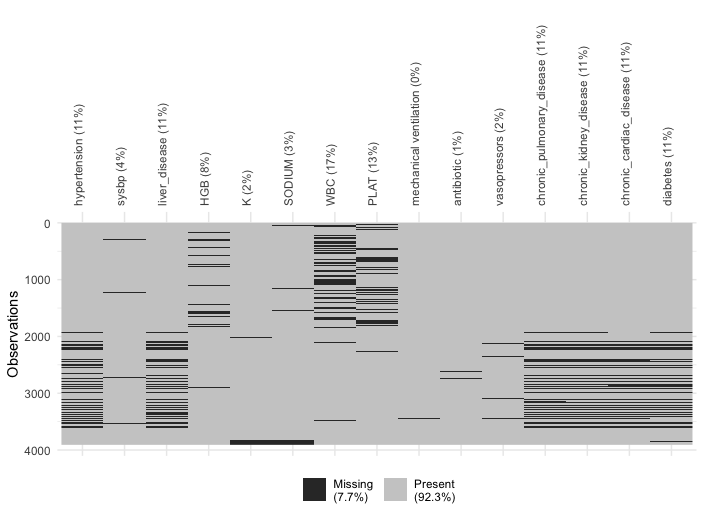


Supplementary Figure 1: Missing values imputed for logistics regression

HGB: hemoglobin; K: potassium; WBC: white blood cell count; PLAT: Platelets.

Supplementary Table 1 – Strengthening the Reporting of Observational Studies in Epidemiology (STROBE) (See STROBE file uploaded)

|  | **Item No.** | **Recommendation** | **Page No.** | **Relevant text from manuscript** |  |  |  |
| --- | --- | --- | --- | --- | --- | --- | --- |
|  |  |  |  |  |  |  |  |
| **Title and abstract** | 1 | (*a*) Indicate the study’s design with a commonly used term in the title or the abstract | 1 | Title |  |  |  |
|  |  | (*b*) Provide in the abstract an informative and balanced summary of what was done and what was found | 2 | Abstract |  |  |  |
| **Introduction** | | |  |  |  |  |  |
| Background/rationale | 2 | Explain the scientific background and rationale for the investigation being reported | 3 |  |  |  |  |
| Objectives | 3 | State specific objectives, including any prespecified hypotheses | 3 | We hypothesized that… |  |  |  |
| **Methods** | | |  |  |  |  |  |
| Study design | 4 | Present key elements of study design early in the paper | 4-6 | The findings in this paper utilized the ISARIC COVID-19 global database. |  | | |
| Setting | 5 | Describe the setting, locations, and relevant dates, including periods of recruitment, exposure, follow-up, and data collection | 4 | Study Design and Study population |  |  |  |
| Participants | 6 | (*a*) *Cohort study*—Give the eligibility criteria, and the sources and methods of selection of participants. Describe methods of follow-up | 4 | Inclusion & Exclusion heading |  |  |  |
|  |  | *Case-control study*—Give the eligibility criteria, and the sources and methods of case ascertainment and control selection. Give the rationale for the choice of cases and controls |  |  |  |  |  |
|  |  | *Cross-sectional study*—Give the eligibility criteria, and the sources and methods of selection of participants |  |  |  |  |  |
|  |  | (*b*) *Cohort study*—For matched studies, give matching criteria and number of exposed and unexposed |  |  |  |  |  |
|  |  | *Case-control study*—For matched studies, give matching criteria and the number of controls per case |  |  |  |  |  |
| Variables | 7 | Clearly define all outcomes, exposures, predictors, potential confounders, and effect modifiers. Give diagnostic criteria, if applicable | 5-6 |  |  |  |  |
| Data sources/ measurement | 8* | For each variable of interest, give sources of data and details of methods of assessment (measurement). Describe comparability of assessment methods if there is more than one group |  | Supplementary table 2 and table 3. |  |  |  |
| Bias | 9 | Describe any efforts to address potential sources of bias | 11 | …we examined in a multivariable model the relationship between baseline characteristics comparing subjects included and those excluded due to missing multiple serum creatinine. |  |  |  |
| Study size | 10 | Explain how the study size was arrived at | 18 | Figure 1 |  |  |  |
| Quantitative variables | 11 | Explain how quantitative variables were handled in the analyses. If applicable, describe which groupings were chosen and why | 6 | Age was categorised into 10-year bins starting from 30 years of age… |  |  |  |
| Statistical methods | 12 | (*a*) Describe all statistical methods, including those used to control for confounding | 6 |  |  |  |  |
|  |  | (*b*) Describe any methods used to examine subgroups and interactions | 6 |  |  |  |  |
|  |  | (*c*) Explain how missing data were addressed | 6 | Variables with more than 20% missingness were excluded from the analysis. Missing values for other variables included were imputed using multiple imputations by chained equation… |  |  |  |
|  |  | (*d*) *Cohort study*—If applicable, explain how loss to follow-up was addressed |  |  |  |  |  |
|  |  | *Case-control study*—If applicable, explain how matching of cases and controls was addressed |  |  |  |  |  |
|  |  | *Cross-sectional study*—If applicable, describe analytical methods taking account of sampling strategy |  |  |  |  |  |
|  |  | (*e*) Describe any sensitivity analyses | 9 | Sensitivity analysis was carried out to examine… |  |  |  |
| **Results** | | |  |  |  |  |  |
| Participants | 13* | (a) Report numbers of individuals at each stage of study—eg numbers potentially eligible, examined for eligibility, confirmed eligible, included in the study, completing follow-up, and analysed | 7 | Figure 1 |  |  |  |
|  |  | (b) Give reasons for non-participation at each stage | 7 | Figure 1 |  |  |  |
|  |  | (c) Consider use of a flow diagram | 18 | Figure 1 |  |  |  |
| Descriptive data | 14* | (a) Give characteristics of study participants (eg demographic, clinical, social) and information on exposures and potential confounders | 22 - 24 | Table 1 | |  |  |
|  |  | (b) Indicate number of participants with missing data for each variable of interest | 22 - 24 | Table 1 |  |  |  |
|  |  | (c) *Cohort study*—Summarise follow-up time (eg, average and total amount) |  |  |  |  |  |
| Outcome data | 15* | *Cohort study*—Report numbers of outcome events or summary measures over time |  |  |  |  |  |
|  |  | *Case-control study—*Report numbers in each exposure category, or summary measures of exposure |  |  | | |  |
|  |  | *Cross-sectional study—*Report numbers of outcome events or summary measures |  |  |  |  |  |
| Main results | 16 | (*a*) Give unadjusted estimates and, if applicable, confounder-adjusted estimates and their precision (eg, 95% confidence interval). Make clear which confounders were adjusted for and why they were included | 7 – 9  25-28 | Table 2 |  |  |  |
|  |  | (*b*) Report category boundaries when continuous variables were categorized | 22 - 24 | Table 1 |  |  |  |
|  |  | (*c*) If relevant, consider translating estimates of relative risk into absolute risk for a meaningful time period |  |  | | |  |

Supplementary Table 2

| **Table S2.** Definitions used for clinical COVID-19 | |  |
| --- | --- | --- |
| Source of definition | Definition for clinical COVID-19 |  |
| World Health Organization (WHO) | A combination of acute fever and cough, |  |
|  |  |  |
|  | Or |  |
|  |  |  |
|  | A combination of three or more of: fever, cough, general weakness and fatigue, headache, myalgia, sore throat, coryza, dyspnoea, anorexia, nausea and vomiting, diarrhoea, altered mental status |  |
| Centers for Disease Control (CDC), United States | At least two of: fever, chills*, rigors *, myalgia, headache, sore throat, new olfactory and taste disorder, |  |
|  |  |  |
|  | Or |  |
|  |  |  |
|  | At least one of: cough, shortness of breath, difficulty breathing* |  |
| Public Health England | New cough, or temperature 37.8°C, or a loss or change in sense of smell or taste |  |
|  |  |  |
| European Center for Disease Prevention and Control | At least one of: cough, fever, shortness of breath, sudden onset anosmia, ageusia or dysgeusia |  |
| * Symptom information not collected in the Case Report Form (CRF) | |  |

Supplementary Table 3 – Comprehensive definitions for comorbidities, complication and outcomes

| Comorbidity | Definition |
| --- | --- |
| Chronic cardiac disease | Any of coronary artery disease, heart failure, congenital heart disease, cardiomyopathy, rheumatic heart disease. |
| Hypertension | Elevated arterial blood pressure diagnosed clinically, >140mmHg systolic or >90mmHg diastolic. |
| Chronic pulmonary disease (not asthma) | Any of chronic obstructive pulmonary disease (chronic bronchitis, chronic obstructive pulmonary disease (COPD), emphysema), cystic fibrosis, bronchiectasis, interstitial lung disease, pre-existing requirement for long term oxygen therapy. |
| Chronic kidney disease | Clinician-diagnosed chronic kidney disease, chronic estimated glomerular filtration rate < 60 mL/min/1.73m^2^, history of kidney transplantation |
| Obesity | Patients for whom an attending clinician has assessed them to be obese - ideally but not necessarily with an objective measurement of obesity, such as calculation of the body mass index (BMI of 30 kg/m^2^ or more) or measurement of abdominal girth. |
| Liver disease (mild, moderate & severe) | Cirrhosis with or without portal hypertension or chronic hepatitis, with or without bleeding or a history of variceal bleeding |
| Type 2 Diabetes | Clinician diagnosed requiring oral or subcutaneous treatment |
| Dementia | Clinical diagnosis of dementia |
| Malnutrition | Any clinically identified deficiency in intake, either of total energy or of specific nutrients that led to a dietetic intervention or referral prior to the onset of COVID-19 symptoms. Does not include people who needed supplementary nutrition solely due to reduced intake during their current illness episode. |
| Complication | |
| Bacterial pneumonia | Clinically or radiologically diagnosed bacterial pneumonia (including community, hospital and ventilator acquired) managed with antimicrobials. Bacteriological confirmation not required. |
| Cardiac arrest | Sudden cessation of cardiac activity with no normal breathing and no signs of circulation. |
| Coagulation disorder | Abnormal coagulation identified by abnormal prothrombin time or activated partial thromboplastin time. Disseminated intravascular coagulation (DIC; consumption coagulopathy; defibrination syndrome) is defined by thrombocytopenia, prolonged prothrombin time, low fibrinogen, elevated D-dimer and thrombotic microangiopathy. |
| Rhabdomyolysis | Rhabdomyolysis is a syndrome characterised by muscle necrosis and the release of myoglobin into the blood. Muscle biopsy, electromyography, radiological imaging and the presence of myoglobinuria are not required for the diagnosis. |
| Acute kidney injury | Acute kidney injury is defined as any of: |
|  | Increase in serum creatinine by ≥0.3 mg/dL (≥26.5 μmol/L) within 48 hours |
|  | Increase in serum creatinine to ≥1.5 times baseline, which is known or presumed to have occurred within the prior 7 days |
|  | Urine volume <0.5 mL/kg/hour for 6 hours |
| Outcomes | |
| ICU admission | Admission to an intensive care unit (ICU) or high dependency care unit (HDU) |
| Invasive mechanical ventilation | Any mechanical ventilation delivered following intubation or via a tracheostomy. Does not include patients who are breathing independently via a tracheostomy. |
| Length of stay | Number of days in hospital up until discharge or study censoring date |
| Still in hospital | Patient is still in hospital at the time of the study censoring date |
| Transferred | Patient has been transferred to another facility that provides medical care. This could be a specialist center for more intensive treatment or a step-down for rehabilitation. It does not include facilities that solely provide social care (these patients should be listed as discharged alive). |
| Discharged alive | Patient has been discharged to their usual place of residence before their illness, to the home of a relative or friend, or to a social care facility, because their illness is no longer severe enough to warrant treatment in a medical facility. |
| Death | Patient died in the hospital |

Supplementary Table 4 – Multivariable Model between baseline and inclusion criteria

|  | **Dependent variable** | | |
| --- | --- | --- | --- |
| *Predictors* | *Odds Ratios* | *CI* | *p* |
| (Intercept) | 0.42 | 0.34 – 0.53 | **<0.001** |
| age y | 1.00 | 0.99 – 1.00 | **0.003** |
| sex [M] | 1.13 | 1.05 – 1.22 | **0.002** |
| sex [U] | 1.55 | 0.51 – 4.73 | 0.441 |
| variant [pre-omicron] | 1.78 | 1.62 – 1.96 | **<0.001** |
| death | 0.99 | 0.84 – 1.18 | 0.944 |
| los3h | 1.00 | 1.00 – 1.00 | 0.346 |
| discharged | 0.50 | 0.42 – 0.58 | **<0.001** |
| Observations | 14107 | | |
| R^2^ Tjur | 0.038 | | |
