# Supplementary material for: Omicron surge impact on acute kidney injury in ICU patients: A study using the ISARIC COVID-19 database
Source: PLoS One. 2025 Nov 20;20(11):e0336843. doi: 10.1371/journal.pone.0336843 (PMC12633887; doi:10.1371/journal.pone.0336843)
Supplement: Attachment — Submitted filename: comments for PlosOne.docx [file pone.0336843.s003.docx]

Firstly, I would like to thank the editor and authors for giving me the opportunity to review the manuscript.

Below is our only comment regarding the latest version submitted.

Introduction section

A single-centre study revealed a three-fold higher prevalence of AKI among intensive care unit (ICU) patients infected with the Omicron versus the Delta variant. Another study identified significant higher prevalence of AKI in the 4th wave which corresponds to the Omicron period [13].

I would like to thank the authors for taking our suggestions into account and adding the recommended reference. However, they should amend the text to state that, in this study, the prevalence of AKI during the fourth wave of the pandemic was lower than in previous waves.
