# Supplementary material for: Omicron surge impact on acute kidney injury in ICU patients: A study using the ISARIC COVID-19 database
Source: PLoS One. 2025 Nov 20;20(11):e0336843. doi: 10.1371/journal.pone.0336843 (PMC12633887; doi:10.1371/journal.pone.0336843)
Supplement: Attachment — Submitted filename: AKI COVID omicron Plos one Round 2.docx [file pone.0336843.s005.docx]

I would like to thank the editor and authors for the opportunity to review this very interesting manuscript on the Omicron Impact on Acute Kidney Injury in ICU Patients.

All comments have been addressed and the manuscript could be accepted for publication

Sincerely yours
